# *HomeSafe* : Postpartum Hypertensive Care Enabled by Electronic Health Record Digital Blood Pressure Capture and Population Health Management

**DOI:** 10.1055/a-2807-4609

**Published:** 2026-03-12

**Authors:** Saba Berhie, Erin Harper, Tooba Anwer, Seema Gupta, David Cantonwine, Sahara Suliman, Ann C. Celi, Khady Diouf, Ellen W. Seely, Louise Wilkins-Haug

**Affiliations:** 1 Maternal Fetal Medicine Division, Department of Obstetrics, Gynecology and Reproductive Biology, Massachusetts General Brigham, Boston, Massachusetts, United States; 2 Department of OBGYN, Maternal Fetal Medicine, Emory University School of Medicine, Atlanta, Georgia, United States; 3 Department of Obstetrics and Gynecology, Emory University School of Medicine, Atlanta, Augusta, Georgia, United States; 4 Division of Endocrinology, Department of Medicine, Massachusetts General Brigham, Boston, Massachusetts, United States; 5 Division of General Medicine and Primary Care, Division of Women's Health, Department of Medicine, Massachusetts General Brigham, Boston, Massachusetts, United States; 6 Department of Obstetrics, Gynecology and Reproductive Biology, Massachusetts General Brigham, Boston, Massachusetts, United States

**Keywords:** hypertensive disease of pregnancy, postpartum, Epic, blood pressure, preeclampsia

## Abstract

**Objective:**

The objective is to present stepwise refinements to *HomeSafe* , an Epic-tethered, population-health-coordinated postpartum pathway for blood pressure (BP) surveillance after hypertensive disease of pregnancy (HDP).

**Study Design:**

*HomeSafe* was designed for HDP people identified during their delivery hospitalization. The program incorporated prepopulated orders for home BP measurement and submission through a smartphone application linked to the electronic health record (Epic). A population health coordinator (PHC) was integrated at Year 2 to support registry tracking, expanded digital support and metric-driven reviews. Study data were managed using REDCap (Research Electronic Data Capture); a secure, web-based application hosted by the Massachusetts General Brigham Digital Research Applications team. Year-to-year analyses were performed for BP submission and route, predictors of timely BP return (≥1 BP in 7 days), 6-week postpartum visit attendance, and clinical and demographic variables.

**Results:**

Across 24 months, 640 postpartum HDP individuals were enrolled; 68.6% (439/640) submitted BP in a timely fashion. In Year 1, 44.9% of BPs submission was by Epic (44.9%), portal (8.2%), or phone (15.5%). No BP was submitted by 31.4%. With a PHC (Year 2), Epic-routed capture increased to 56.5%, and phone/portal-dependent routes decreased to 11.6% ( *p* = 0.0006). Program enrollment increased from 245 to 485 ( *p* < 0.001) without changes in delivery volume. Independent negative predictors of BP return were Black non-Hispanic race, public insurance, and multiparity; HDP subtype, delivery mode, antihypertensive use, and neonatal intensive care unit admission were not predictive. *HomeSafe* engagement strongly predicted 6-week postpartum visit attendance (89.9 vs. 65.4%, *p* < 0.0001).

**Conclusion:**

*HomeSafe* , an EHR-tethered, postpartum BP surveillance pathway, when partnered with a population health management approach and a coordinator provides significant improvements in BP ascertainment, enrollment scalability, and 6-week postpartum engagement. Persistent disparities by race and insurance status highlight a need for equity-focused approaches.

**Key Points:**

- *HomeSafe—* Epic-linked monitoring of postpartum blood pressure.
- Population health tenets improve postpartum surveillance.
- Gaps in postpartum surveillance of high-risk person remain.

## Introduction

Hypertensive disease of pregnancy (HDP) is a powerful predictor of increased morbidity and mortality during the first year postpartum (PP). 1 2 3 HDP is associated with an increased risk of stroke, chronic hypertension, and hospital readmission during the PP period. 4 5 In the United States, HDP occurs in approximately 14% (1 in 7) pregnancies with increased prevalence in persons over 35 years of age, those indicating race as Black, non-Hispanic and those in the lowest income quartile based on zip codes. The prevalence of HDP continues to increase globally and care recommendations for those following HDP vary and are an active area of investigation. 6 7

Given these risks following HDP, the American College of Obstetricians and Gynecologists (ACOG) recommends blood pressure (BP) evaluation at 72 hours and within 7 to 10 days after delivery for severe and non-severe disease, respectively. 6 8 Toolkits for PP-HDP management recently became available from the American College of Cardiology and are paired with a review of the clinical approaches. 9 10 Engaging this specific population to continue health care following delivery faces challenges, including the conflicting priorities of caring for a newborn, care for other children, return to work, identification as a healthy individual, and lack of prior care engagement with their primary care provider. The successful transition to primary care is identified as gap to close by a recent Society of Maternal Fetal Medicine Special Statement. 11

Various approaches for PP-HDP surveillance are emerging, including remote BP monitoring (RBPM) shown to be as clinically effective as office-based measurement in the number of PP readmissions and acquisition of BP within 1 week. 12 RBPM is often supplemented by virtual visits, community health worker engagement, phone calls, text messaging, as well as patient EHR portal reminders. Despite differing management strategies with RBPM, systematic reviews support RBPM as a reliable method to reduce PP readmission, lower average BP within the first year PP and sustain lower BP through several years follow-up. 13 14 For instance, one randomized controlled trial found that BP ascertainment within 10 days' PP was significantly greater in RBPM program participants compared with those receiving standard care. 15 In the Self-Management of Postnatal Antihypertensive Treatment Trial (SNAP-HT), women randomized to an RBPM program had consistently lower diastolic BPs than those receiving standard care, a statistically significant difference that lasted for up to 4 years' PP. 16 17 While encouraging, integration into existing clinical practice to manage PP persons following HDP and transitioning them to primary care remains a significant challenge and substantial variation exists. 18 19

In the nonpregnant population, population health management (PHM) of “at risk cohorts” such as adults with hypertension or diabetes is recognized as a sustainable and scalable approach. Built around the clinical integration of a systematic approach of registry maintenance, coordinators, digitalization and algorithmic management, over the past decade, PHM for nonpregnant cohorts successfully transitioned from research to clinical implementation to integration in reimbursement policies. PHM is centered on a proactive approach to a subpopulation's specific needs with a goal to improve their health. 20 21 22 Automation, team approaches and ongoing metric analyses are heralded as key components. 23 24 A recent, successful implementation for cardiovascular disease risk factor management among a large, diverse veteran population is represented by TEAMS—Team-supported, electronic health record leveraged, active management. 25 A population health coordinator (PHC) reviewed patient data and used an algorithm to identify and communicate with patients who met criteria for uncontrolled BP and then followed a multistep process to engage with these patients. This program found the high-touch engagement with a PHC improved patient adherence with scheduled primary care appointments and engagement with their hypertension care.

Leveraging insights from PHM and, in particular, work from programs designed for hypertension in the nonpregnant, diverse populations, we created a PP hypertension management approach. 25 26 Our strategy incorporated PHM principles and its four tools: (1) registry management by a population health care coordinator, (2) RBPM conveyed through a smartphone application to an electronic medical record (Epic), (3) multidisciplinary teams, and (4) a stepwise and metric-based approach to evaluate barriers to transmission of BPs. The program, termed *HomeSafe* was launched at an academic medical center in January 2023 and its development and 2 years of metric-based iterations are presented here.

## Materials and Methods

### Study Cohort

*HomeSafe* is partially funded by the Massachusetts Department of Public Health—Health and Human Services Award (March 6, 2023–June 29, 2027). “The MA Maternal Health Innovation and Data Capacity Program” aimed at addressing the delivery to 6-week PP care gap following HDP. A second phase of the program is planned to assess risk stratification and transition from obstetric to primary care following 6 weeks' PP.

The *HomeSafe* cohort included PP people diagnosed during their delivery hospitalization with HDP: chronic hypertension, chronic hypertension with superimposed preeclampsia, gestational hypertension, preeclampsia with severe features, and/or preeclampsia without severe features at Brigham and Women's Hospital (BWH) in Boston, Massachusetts. HDP diagnoses were assigned by the physician-led PP team and could have occurred at any point during their antenatal and delivery care. BWH is an academic medical center with an annual delivery volume of approximately 6,000 live births per year.

Eligible patients (∼2,100 persons of the 6,000 total births per year) were those who received obstetric care through the faculty practice at BWH, which includes Maternal–Fetal Medicine (MFM) faculty and fellow clinics, academic specialist obstetric clinics, and resident-led obstetric clinics. These patients all receive care from a single inpatient PP obstetric rounding team comprised of a physician assistant from the MFM or OBGYN practice, OBGYN resident physicians, and a supervising attending physician from the MFM or academic specialist practice. Patients who received prenatal care from the certified nurse midwife practice or a private obstetrician and were transferred to the faculty physician group for hypertensive disease, were also eligible. These groups were selected for rollout of the program due to the consistency of the rounding team.

### Recruitment and Enrollment

Eligible patients were identified during PP rounds by the inpatient MFM-OB clinical team at BWH. This team of PP rounders introduced *HomeSafe* care to the HDP PP persons, assured smartphone availability, internet access, and placed a prepopulated order in the electronic health record (Epic), which enrolled the individual in the program. Enrolled participants indicated English or Spanish as primary languages, and agreed to self-directed BP monitoring. Participants were already active on the patient portal in Epic and its message communication program (Patient Gateway) and familiar with its use through their prenatal care. Participants were included regardless of their need for antihypertensive therapy during the PP period. Individuals were excluded if they planned to receive PP care outside the Brigham and Women's healthcare system or if they developed new-onset hypertension after hospital discharge.

This paper reports on participants who were enrolled during the first 24 months of implementation: January 1, 2023, through December 31, 2024.

### Blood Pressure Review and Management Pathways

The *HomeSafe* outpatient RN team reviewed BP submissions through Epic flowsheets daily Monday through Friday (8 a.m.–4 p.m.). Epic-generated high-value BPs alerts (systolic >160 mm Hg or diastolic >100 mm Hg) were received by the *HomeSafe* team of RNs. A provider (RN or Physician assistant) telephoned the PP person to assess symptoms and initiate or adjust antihypertensive therapy using a team adjudicated treatment algorithm ( Supplementary Figs. S1 and S2 [available in online version]). The Epic program also incorporated a visual alert to the PP person to call the RN during office hours to discuss elevated BP. During off-hours (weekends and after 5 p.m. on weekdays), patients were instructed to page the on-call obstetric provider if they received an alert or had hypertension concerns. 24-hour care coverage was available through either in-person triage or labor and delivery.

For patients not on antihypertensive medications, home BP monitoring was encouraged once a day for 6 weeks' PP. If values remained within desired range (<140/90), patients were discharged from the program at 2 weeks' PP with instructions to continue daily, self-monitoring independently until their 6-week visit. They were provided guidance on contacting the clinic or on-call obstetrician for increases in BPs, or symptoms such as headache, vision changes, and to arrange an appointment with their primary care provider.

For those on antihypertensive therapy, medication titration was guided by a provider-adjudicated algorithm. When medications were discontinued, BP monitoring continued until 6 weeks' PP before program discharge. If BP was not at goal during this time, medications were adjusted guided by the algorithm. Close MD or physician assistant follow-up continued until target values were achieved or the individual had transitioned to a primary care or internal medicine provider.

All participants were discharged from the program at 6 weeks' PP.

### Stepwise, Operational Adjustments to *HomeSafe*

**Fig. 1:**
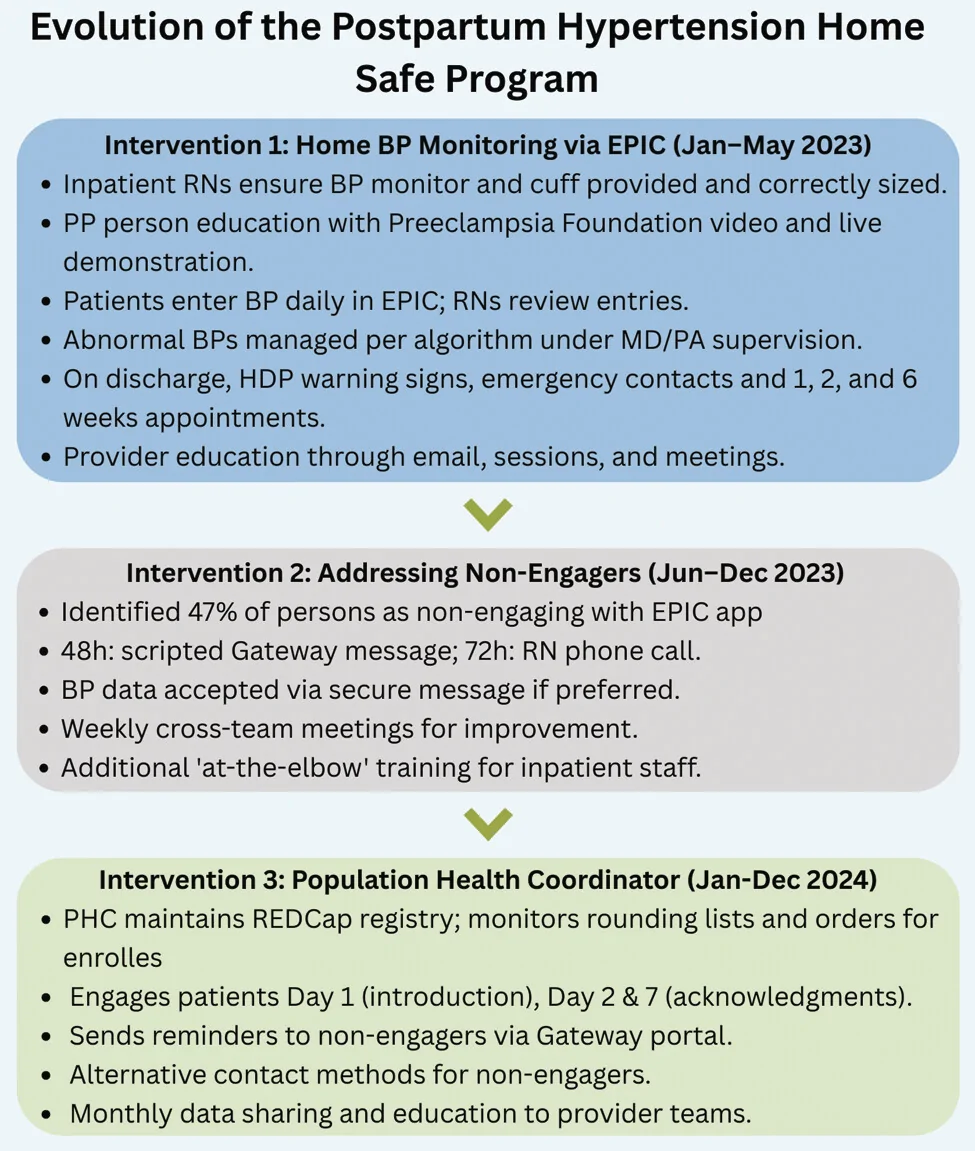
Stepwise development of population health management approach to postpartum management following hypertensive disease of pregnancy— *HomeSafe* .

The *HomeSafe* program implemented stepwise changes with each subsequent intervention being team-driven and building upon the foundation of the previous metrics ( Fig. 1 ).

(1) Introduction of remote home blood pressure monitoring via Epic app (January 2023 through May 2023)

Enrollment occurred as above. Inpatient PP nurses insured that patients had or were ordered to receive a BP monitor and cuff prior to discharge, provided education as to the monitor's use and provided a link to the smartphone Epic application. BP cuffs were correctly sized to the patient by the nurse. BP cuffs and monitors ordered were either the basic model Omron or A&D dependent on the person's insurance coverage. The inpatient nurse taught the individual how to use the monitor and then the patient demonstrated appropriate use back to the nurse. A video demonstrating proper technique for using an automated BP monitor developed by the Preeclampsia Foundation was provided. The inpatient nursing team assured the individual could access the Epic application and values entered were reviewed by the PP nursing team daily until discharge. Instructions included continued daily entry of BPs after discharge for a minimum of 1 week. All enrolled patients were informed about warning symptoms of high BP such as headache, visual change, or right upper quadrant pain and were given contact numbers for their OB healthcare providers and the emergency line for weekend and night calls. All *HomeSafe* participants were scheduled for follow-up visits (either virtual or office) at 1, 2, and 6 weeks' PP. BPs submitted through the Epic app were reviewed by the *HomeSafe* team of three outpatient RNs. Management decisions of BP elevations were guided by an algorithm under the oversight of the supervising provider (MD or physician assistant).

Education regarding the program start and its content was given to providers of PP care at all clinical levels (MD, physician assistants, RNs) in both the inpatient and outpatient setting through email announcements, small group educational sessions, and monthly meetings with the team (MD supervisor, inpatient and outpatient RN representatives, and physician assistants).

(2) Addressing Nonengagers with the Epic App (June 2023 through December 2023)

At 6 months from program start, 47.2% of persons had not engaged with the Epic app and were without a BP submission during their first week after delivery discharge. After this scheduled review, the outpatient RN team initiated systematic patient follow-up if BP measurements were not received at 48 hours (Patient Gateway portal message) or by 72 hours (by telephone call). Telephone calls were noted in the Epic chart by the RN. PP persons were allowed to return BPs by secure patient portal or on the telephone call, if they preferred and these were documented in a note.

At this 6-month review, the interdisciplinary outpatient team provided additional at-the-elbow, ongoing educational support to the inpatient providers (RNs, physician assistants, and MDs) about the program and its processes. The outpatient team initiated weekly meetings centered around challenges, process improvement, workload, and continued education to the rounding clinicians and inpatient RNs.

(3) Introduction of a Population Health Coordinator (January 2024 through December 31, 2024)

Review at 1 year identified a loss of standardization of person's PP-BP submission as a wider clinical provider team assumed care. Reversion to prior practices of telephone or patient portal interaction occurred. To address these concerns, tools of PHM programs were reviewed and a PHC was introduced. The PHC's role was 4-fold to match the underpinnings of PHM—(1) registry maintenance, (2) expanding Epic automation through scripted patient portal messages ( Supplement 3 , available in the online version), (3) coalescing the *HomeSafe* team as a point person, and (4) metric assessment to refine the program. The PHC provided registry development (REDCap) and maintenance with daily surveillance of the PP rounding lists, review of orders placed and through direct communication with the PP rounding teams. 27 28 To expand digital capacities integral to the Epic system, the PHC also initiated structured communication with *HomeSafe* enrollees—on day 1 postdischarge to reinforce the rationale for continued surveillance, the team approach to their ongoing care, and the expectation of daily BP measurements on their Epic-enabled application for 2 weeks. The PHC continued communication and acknowledged normal BP (Days 2 and 7). Lastly, the PHC sent scripted reminders by the Gateway patient portal to those who had not returned BP measurements (Days 2 and 4 posthospital discharge). If no BPs were submitted by 6 days, patients were contacted by an RN with a phone call logged in the patient chart. Patients were also contacted by phone call if they stopped uploading BPs within the first 2 weeks. Nonengagers with the Epic app were asked by Patient Gateway for alternative means of communication. As a representative of the *HomeSafe* team, the PHC also served as an additional continuity touchpoint for PP persons for nonurgent, hypertension needs (delegated to RNs as needed) and for providers needing assistance with enrollment. Lastly, the PHC was instrumental in maintenance of the *HomeSafe* registry, which provided a platform for metric analysis at regular intervals, providing ongoing assessment of engagers and nonengagers to identify future approaches to care, as well as oversight of the program. REDcap can be queried for ongoing metrics as well as addressing gaps in care.

Continued education about *HomeSafe* and the addition of a PHC was provided to the clinical providers at monthly faculty division meetings, monthly clinical office meetings and through direct contact with the resident teams who changed every 6 weeks. Pilot data were shared at these meetings and operational feedback was incorporated.

Stepwise iterations of the *HomeSafe* Program over 2 years are depicted in Fig. 1

### Data Collection and Statistical Assessment

Abstraction of demographics, program participation, and clinical care during the 6 weeks' PP were provided under MGB-BWH IRB 2023P001435. Maternal demographics including determinants of health care utilization (including, but not limited to, insurance status, race/ethnicity, marital status, parity) and clinical characteristics (including, but not limited to, maternal comorbidities, mode of delivery, gestational age at delivery, subcategorization of HDP and antihypertensive medication use) were examined by calculating the mean, standard deviation or *n* (%) in the overall cohort. We then reported both the distribution of HDP diagnoses and the overall distribution *n* (%) of method of how BP measurements were sent within 7 days' PP. Next, we compared the demographic and clinical characteristics of those that reported BP measurements within 7 days' PP and those that did not by using Wilcoxon, Fisher's exact or chi-squared tests where appropriate. Multivariate logistic regression models were used to compare demographic and clinical characteristics between individuals who reported a BP measurement within 7 days' PP and those who did not. Variable selection was performed using both forward and backward stepwise procedures (based on *p* < 0.20) to retain predictors that best explained the outcome. This same approach was utilized in separate logistic regression models comparing demographic and clinical characteristics between those who sent BP measurements through Epic within the first 2 days' PP. Fisher's exact or chi-squared tests were used to compare how BP measurements were received within the first 7 days' PP before the involvement of a PHC and after, as well as if participants were readmitted PP or length of stay of readmission was greater than 24 hours. Statistical analyses were performed using R version 4.5.1.

## Results

**Fig. 2:**
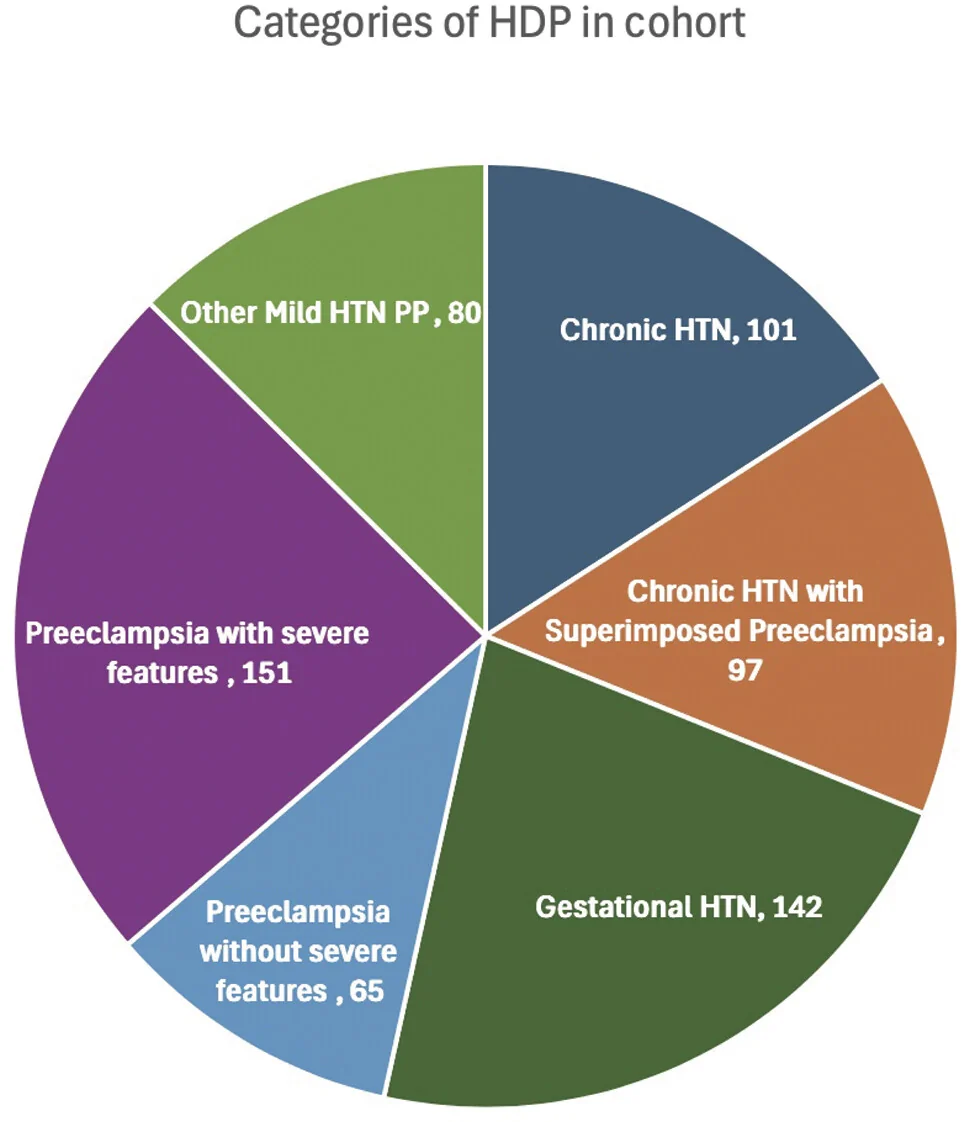
Distribution of hypertensive disease of pregnancy among *HomeSafe* enrollees.

Over the 2 years, PP persons ( *N* = 640) were enrolled during their delivery hospitalization with a diagnosis of HDP. The population was racially and ethnically heterogeneous ( Table 1 ). HDP subcategories were evenly spread across all subtypes ( Fig. 2 ). Antihypertensive medication was utilized by 20.5% of persons during pregnancy and 65.5% had antihypertensive and/or diuretic agents started prior to delivery discharge.

**Table 1:** Maternal demographics among those *HomeSafe* participants enrolled from 2023 to 2024

Population characteristics	Study population ( *N* = 640)
	Mean (SD) or *N* (%)
Maternal age at delivery (y)	33.3 (5.8)
Maternal race/ethnicity	
White/non-Hispanic	286 (44.7%)
Black/non-Hispanic	140 (21.9%)
Asian	36 (5.6%)
Hispanic	143 (22.3%)
Other/mixed	16 (2.5%)
Unknown	19 (3.0%)
Marital status	
Married	363 (56.7%)
Single	240 (37.5%)
Other	31 (4.8%)
Unknown	6 (0.9%)
Insurance status	
Private/HMO	491 (76.7%)
MassHealth/Medicaid/self-pay	147 (23%)
Missing	2 (0.3%)
Primiparous	351 (54.8%)

Abbreviations: HMO, Health Maintenance Organization; SD, standard deviation.

### Submission of Blood Pressure Measurements

Over the 2 years, receipt of at least one BP within 7 days of discharge was obtained in *N* = 439 (68.6%) persons. Biannual assessment of data indicated BP return rates within 7 days ranged from 65.0% to 71.0%, without significant variation over the 2 years.

The submitted BPs within 7 days were distributed as 44.2% through the Epic app without prompting by Day 2 with additional 9.2% sent to the Epic app after a Day 2 reminder (total = 53.4% through Epic app). An additional 15.1% of BP measurements were retrieved through patient response to a portal reminder or RN telephone call by Day 7.

Returning BP measurements within 7 days was significantly lower by maternal minority race/ethnicity, single marital status, public insurance status, and multiparity. Clinical characteristics including mode of delivery, neonatal intensive care unit admission, subcategory of HDP, did not individually influence whether a BP was received within 7 days ( Table 2 ) In a stepwise multivariant analysis, Black/non-Hispanic, having public insurance, and being multiparous each remained as independent contributors to a lower likelihood of a BP received within 7 days ( Table 3 ).

**Table 2:** Maternal demographics among those *HomeSafe* participants enrolled from 2023 to 2024 stratified on blood pressure measurements received within the first 7 days' postpartum

Population characteristics	BP sent within 7 days' PP, *N* = 436	BP not sent within 7 days' PP, *N* = 204	*p* -Value
	Mean (SD) or *N* (%)	Mean (SD) or *N* (%)	
Maternal age at delivery (y)	33.6 (5.5)	32.7 (6.2)	0.16
Maternal race/ethnicity			
White/non-Hispanic	225 (51.6)	61 (29.9)	<0.0001
Black/non-Hispanic	76 (17.4)	64 (31.4)	
Asian	29 (6.7)	7 (3.4)	
Hispanic	89 (20.4)	54 (26.5)	
Other/mixed	7 (1.6)	9 (4.4)	
Unknown	10 (2.3)	9 (4.4)	
Marital status			
Married	276 (63.3)	87 (42.7)	<0.0001
Single	141 (32.3)	99 (48.5)	
Other	19 (4.4)	18 (8.8)	
Insurance status			
Private/HMO	366 (83.9)	125 (61.3)	<0.0001
MassHealth/Medicaid/self-pay	69 (15.8)	78 (38.2)	
Primiparous	256 (58.7)	95 (46.6)	0.005
Mode of delivery			
Cesarean section	233 (53.0)	113 (55.0)	0.05
Vaginal delivery-assisted (forceps/vacuum)	32 (7.3)	5 (2.5)	
Spontaneous vaginal delivery	171 (39.7)	86 (42.5)	
NICU admission	127 (29.1)	68 (33.3)	0.32
Maternal diagnostic variables			
Hypertension diagnoses			
Chronic hypertension diagnosis	69 (15.8)	32 (15.69)	
Chronic hypertension with superimposed preeclampsia	65 (14.9)	32 (15.7)	
Gestational hypertension diagnosis	108 (24.8)	34 (16.7)	
Preeclampsia without severe features diagnosis	45 (10.3)	20 (9.8)	
Preeclampsia with severe features diagnosis	110 (25.2)	41 (20.1)	
Mild PP-HDP	39 (8.9)	41 (20.1)	0.57

Abbreviations: BP, blood pressure; HDP, hypertensive disease of pregnancy; HMO, Health Maintenance Organization; NICU, neonatal intensive care unit; PP, postpartum.

**Table 3:** Multivariate regression of contribution of maternal demographic characteristics to likelihood of blood pressures being received within the first 7 days' postpartum

	Unadjusted OR (95% CI)	Adjusted OR (95% CI)
Maternal age (y)	1.03 (1.00, 1.06)	
Maternal race/ethnicity		
White/non-Hispanic	Ref.	
Black/non-Hispanic	0.44 (0.30, 0.66)	0.46 (0.29, 0.75)
Asian	1.96 (0.89, 4.94)	
Hispanic	0.69 (0.47, 1.02)	0.64 (0.40, 1.03)
Other/mixed/unknown	0.35 (0.12, 0.94)	0.39 (0.13, 1.16)
Marital status		
Married	Ref.	
Single	0.51 (0.36, 0.71)	0.75 (0.51, 1.11)
Other/unknown	0.73 (0.35, 1.57)	
Public insurance status	0.30 (0.21, 0.44)	0.47 (0.31, 0.72)
Primiparous	1.63 (1.17, 2.28)	1.61 (1.13, 2.32)

Abbreviations: CI, confidence interval; OR, odds ratio.

### Population Health Coordinator

Introduction of the PHC was significantly associated with an increased number of persons enrolled in the program ( *N* = 245 in 2023, vs. *N* = 485 in 2024, *p* < 0.001 without significant change in delivery volumes over the 24 months).

The proportion of individuals returning BPs within 7 days remained the same ( Table 4 ).

**Table 4:** Assessing the role of a population health coordinator in how blood pressures were received within the first 7 days' postpartum

	Epic a	Patient gateway a	Phone call a	BP within 7 d	None received	Total
No public health coordinator January 1–December 31, 2023	110 (44.9%)	20 (8.2%)	38 (15.5%)	168 (68.6%)	77 (31.4%)	245
Public health coordinator January 1–December 31, 2024	223 (56.5%)	22 (5.6%)	26 (6.6%)	271 (68.6%)	124 (31.4%)	395

Abbreviation: BP, blood pressure.

a *p* < 0.05 for distribution of method by which BP was received.

**Fig. 3:**
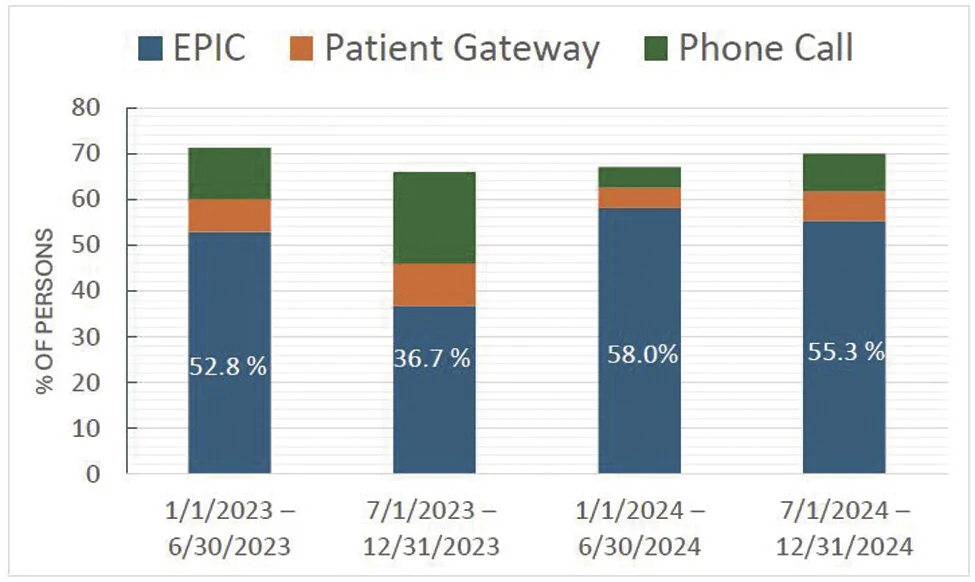
Method of submission of blood pressure measurements within 7 days by postpartum persons to the *HomeSafe* Program before population health coordinator (January 2023–December 2023) and after (January 2024–December 2024).

Introduction of the PHC in Year 2, significantly altered the manner of receipt of the BP ( Fig. 3 ). In Year 1, without the PHC, among the PP persons who engaged with RBPM 65.4% (110/168) of BPs were returned through the Epic app and 34.6% (58/168) through RN staffed phone calls or portal. In Year 2, after introduction of the PHC and initiation of structured Patient Gateway platform messaging, 82.3% (223/271) of BPs arrived significantly more often the Epic app in an automated fashion without the need for individualized, bidirectional communication when BPs were normal ( *p* = 0.01; for Year 1 vs. 2 method of returning BP).

Active participation in *HomeSafe* was tied to the rate of readmission for hypertension. Returning a BP within 7 days was significantly associated with increased chances of readmission for hypertension from 11/201 (5.4%) to 68/435 (15.6%) ( *p* = 0.0004). Those with a BP submission through *HomeSafe* , had shorter PP admissions (<24 hours' inclusive of triage visits; 72.7 vs. 27.3%), although this was not significant.

### 6-Week Postpartum Visit

Attendance at the 6-week PP visit occurred in 82.0% (525/640) and was significantly higher among those who engaged with the *HomeSafe* program through BP submission (392/640, 89.9 vs. 133/640; 65.4% *p* < 0.0001) than those who did not engage. In bivariable analyses, predictors of nonattendence at 6 weeks were nonengagement with *HomeSafe* (unadjusted odds ratio [OR]: 0.21, 95% confidence interval [CI]: 0.14–0.32), public insurance (unadjusted OR: 0.18, 95% CI: 0.12–0.28) and single persons (unadjusted OR: 0.37, 95% CI: 0.24–0.57). The remaining demographic variables of race, ethnicity, and parity did not indicate a significant association with attendance at 6 weeks. Measures reflecting the extent of hypertensive disease showed mixed associations: use of antihypertensive medication during pregnancy was not associated with attendance (unadjusted OR: 1.27, 95% CI: 0.75–2.15), whereas PP outpatient initiation of antihypertensive therapy was associated with higher attendance in unadjusted analyses (unadjusted OR: 1.94, 95% CI: 1.13–3.33).

Multivariable logistic regression of these same demographics and disease severity variables, indicated nonengagement with *HomeSafe* and public insurance remained statistically independently associated with nonattendance at the 6-week PP visit, (nonengagement in *HomeSafe* 0.30 [0.19–0.49]; public insurance adjusted OR: 0.28 [0.17–0.47], both *p* < 0.001).

## Discussion

Our 2-year (2023–2024) *HomeSafe* program for the management of HDP-PP persons enrolled a large number of persons with diverse demographics ( *N* = 640). Consistently through the 2 years of the program, 68.8% of HDP-PP persons submitted BPs within the first 7 days. This is similar to the 60 to 90% submission rate across a variety of RBPM platforms recently reviewed from the literature. 29 Additionally, our stepwise approach to program development provided an opportunity to examine operational details. RBPM following HDP rapidly entered clinical practice, especially during coronavirus disease. Systematic review supports the reliability of home measurements, and patient and provider acceptability, although operational details of programs outside of research structures are less available. 29 Additionally, considerable variability exists in published research cohorts around communication modality including telephone calls, texting, portal messages, Bluetooth-enabled transmission, or a web-based application. Variation is also present in BP management, including individualized clinical judgment, algorithms overseen by clinicians (RNs, MDS, pharmacists), or, in one case, artificial intelligence. 29 An examination of our stepwise, metric-driven alterations to our *HomeSafe* program over 2 years provides further insight into a large-volume program's operational development, highlighting BP submission linked to the individual's electronic medical record, and sustainability of program management through population health principles.

Our program started by providing digital HBPMs at discharge to patients who had a HDP, orienting them to the smartphone app aligned with their electronic medical record (Epic) and asking the patients to send in daily BPs by the app. An Epic-linked app had already been created for collection of BPs in nonpregnant individuals followed in PHM programs and could be modified for the PP person by modifying alert ranges, BP frequency and notification method for nonreceipt of BPs. As part of the electronic medical record, this smartphone app is available to patients and providers without cost. While initiating digital home BP monitoring in PP care is no longer unique, returning the reading to an existing linked electronic health record system is only now emerging. In our demographically heterogeneous and large ( *N* = 640) population, the use of the Epic-embedded smartphone app across the 2 years ranged from 44.9 to 56.9% of the population. Our population's use of the use of the Epic app is lower than the 69 and 70.6% reported engagement for a similar Epic app in smaller cohorts ( *N* = 54 and *N* = 368) though varied definitions for “engagement” exist. 30 31 The *HomeSafe* level of participation is noteworthy as being derived from a larger population PP-HDP persons ( *N* = 640) with rolling enrollment over 2 years. Our findings also reiterate the need for further exploration of the demographic differences in Epic app utilization previously reported. 31 This is of particular relevance as some of the identified demographic characteristics of persons with the least engagement with the Epic app are simultaneously those with higher rates of HDP and its associated morbidity and mortality (such as race and public insurance). Greater attention to digital and health literacy as well as lived experiences provided opportunities for further attention.

To focus on sustainability, we incorporated the principles of PHM, including registry maintenance, digital automation, algorithmic-guided team management, and metric-driven analyses. 23 24 Notably, the 2024 Centers for Disease Control “Hypertension in Pregnancy Change Package” cites PHM as one of its four tenets for managing hypertension in pregnancy. 32 33 Operationally, to optimize clinical provider time (RN, physician assistant, and MD) support care at their highest level of license and also to standardize patient management, the *HomeSafe* program underwent two major iterative changes—during our first 6 months of implementation, distribution of the BP cuffs, Epic app, and management of BPs were standardized, but approaches to those persons who did not engage remained individualized. After reviewing at 6 months, reminders to the nonengagers were initiated by RNs either through telephone calls or patient portal messages. Throughout the first year, the RNs continued to be team contact fielding communications through telephone or medical record portal messages.

A population health model was introduced at the start of the second year. Originating in the outpatient care of nonpregnant adults with hypertension and diabetes, PHM incorporates the principles of registry building and maintenance, digital tools, team approach, and ongoing metric analyses as key components. 23 24 The introduction of the PHM approach successfully shifted PP-HDP home BP monitoring from labor-intensive, provider-dependent encounters toward a systematic, sustainable, and patient-centered model promoted in recent literature. 32 33 34

In year 2 of *HomeSafe* , the PHM approach prioritized patient engagement and short-term BP control embedding a PHC within the care team. 35 With a PHC, a robust patient registry was built and maintained, standardized patient portal reminders automated, and two-way portal communication between PP persons and the PHC-enabled. These changes did not alter the overall submission of BPs within 7 days (68.8%). Notably, however, there was a significant shift toward increased submission through the Epic app (from 44.9 to 56%, pre- and post-PHC, respectively) and an approximately halving of the need for BP transmission through telephone or portal (from 23.7 to 12.2%, pre and post, respectively). Transfer to the PHC of nonengaged patient communication allowed the RNs to concentrate on BP management rather than program engagement. The PHC in Year 2 further solidified the enrollment approach and increasing the number of patients participating. The PHC's systematic registry maintenance, proactive portal communication, and targeted reminders were central to this improvement. Looking ahead, enhanced algorithmic management and expansion of PHC responsibilities—such as appointment scheduling, quarterly outcome reviews, and proactive identification of persistent care gaps—mirror functions of established adult PHM programs and represent natural next steps.

Despite these gains, challenges were still present. The most prominent was the persistence of approximately 1/3 of persons who did not engage with the program through submission of a BP within 7 days. This occurred both during the first year and throughout the second year despite an intervention to systematically engage with HDP-PP persons through standardized portal messaging. Clinical surrogates of disease severity (e.g., HDP subtype, prior antihypertensive use, discharge on medications, or neonatal intensive care unit admission) did not predict submission of BP reading. Instead, multivariable analyses identified demographic factors—Black race, public insurance status, and multiparity—as significant determinants of engagement and BP submission. For the group who did not engage with the Epic app, portal-based reminders proved ineffective, underscoring the need for alternative strategies.

Our findings further highlight that the social determinants of health remain a barrier to care even with a resource-intensive approach to disparity in HDP. 36 Understanding the PP patient experience of HDP will also be essential in thinking of strategies to improve outcomes and adherence to RBPM. 37 Patient-centered approaches, such as surveys, focus groups, or tailored outreach, warrant exploration, particularly as nonengagers demonstrated a trend toward longer rehospitalization when readmitted. On the other hand, engagers had almost a 3-fold higher readmission rates for uncontrolled BP than nonengagers. The explanation may be multifactorial and requires further assessment. PP persons engaged with the program may have been more receptive to the education around the risks of PP hypertension, although the specific category of HDP did not alter the rate of engagement. The return of BP may have allowed those monitoring to identify early concerns requiring more intensive management. Lastly, despite the recommended treatment algorithms, clinical judgement may contribute.

Collectively, these findings demonstrate the feasibility and sustainability of a PHM-based model for PP-HDP care. Ongoing engagement with the patient's obstetric practice could provide an opportunity to build stronger partnerships with primary care providers and should be a part of future work. Given an average interpregnancy interval of 2 years, the PP period represents a critical window to optimize BP control and address comorbidities that influence both recurrence risk and lifelong health. Lessons learned from established PHM programs in adult hypertension and diabetes provide a framework for those who undergo a hypertensive condition during pregnancy. The goals of PHM center around a sustainable, team-driven approach to care grounded in home health monitoring, registry maintenance and metric analysis of gaps in care. With a decade of experience in PHM for nonpregnant adults with chronic hypertension and diabetes, the insights gained on cost efficacy, and program reimbursement should be brought to the PP-HDP population.
