# Supplementary material for: HomeSafe : Postpartum Hypertensive Care Enabled by Electronic Health Record Digital Blood Pressure Capture and Population Health Management
Source: Am J Perinatol. 2026 Mar 12;43(12):1648–56. doi: 10.1055/a-2807-4609 (PMC13503027; doi:10.1055/a-2807-4609)
Supplement: Supplementary file 1 [file 10-1055-a-2807-4609_29438370.pdf]

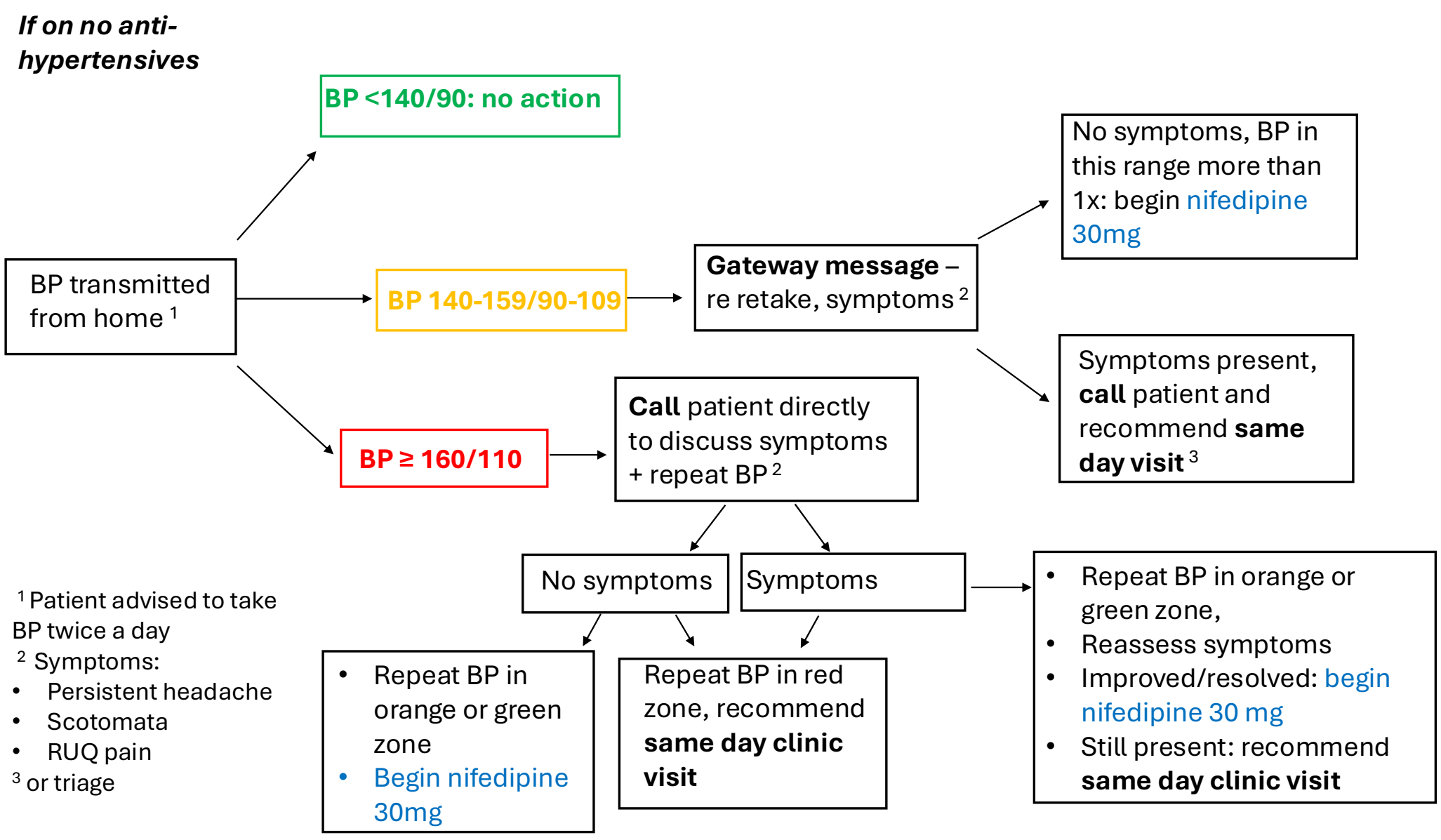
If on no anti-hypertensives
BP <140/90: no action
BP transmitted from home [1]
BP 140-159/90-109
Gateway message – re retake, symptoms [2]
No symptoms, BP in this range more than 1x: begin nifedipine 30mg
Symptoms present, call patient and recommend same day visit [3]
BP ≥ 160/110
Call patient directly to discuss symptoms + repeat BP [2]
No symptoms
Symptoms
Repeat BP in orange or green zone
Begin nifedipine 30mg
Repeat BP in red zone, recommend same day clinic visit
Repeat BP in orange or green zone,
Reassess symptoms
Improved/resolved: begin nifedipine 30 mg
Still present: recommend same day clinic visit
[1] Patient advised to take BP twice a day
[2] Symptoms:
Persistent headache
Scotomata
RUQ pain
[3] or triage

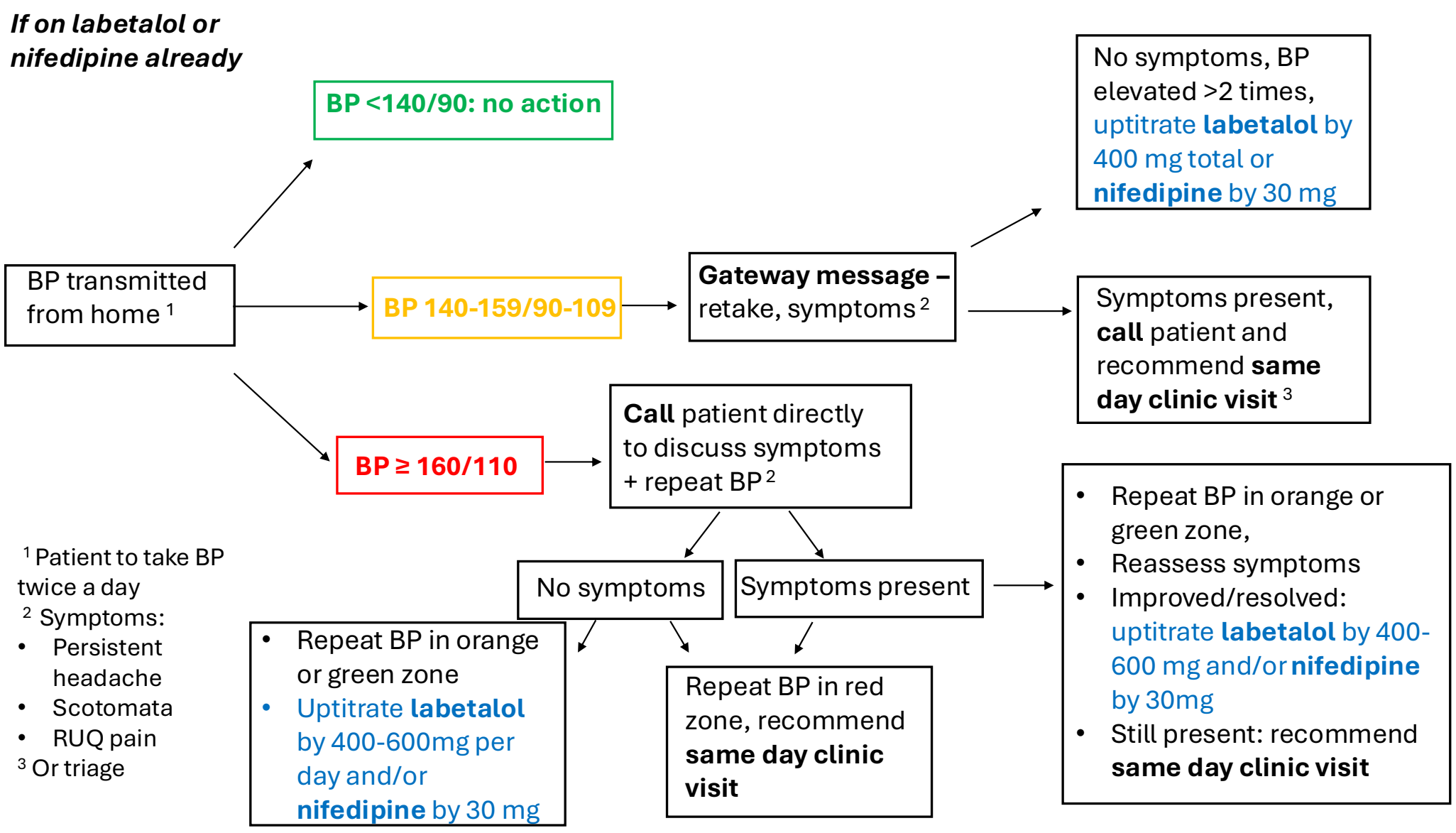
If on labetalol or nifedipine already
BP transmitted from home [1]
BP <140/90: no action
BP 140-159/90-109
BP ≥ 160/110
Gateway message – retake, symptoms [2]
No symptoms, BP elevated >2 times, uptitrate labetalol by 400 mg total or nifedipine by 30 mg
Symptoms present, call patient and recommend same day clinic visit [3]
Call patient directly to discuss symptoms + repeat BP [2]
No symptoms
Symptoms present
Repeat BP in orange or green zone
Uptitrate labetalol by 400-600mg per day and/or nifedipine by 30 mg
Repeat BP in red zone, recommend same day clinic visit
Repeat BP in orange or green zone,
Reassess symptoms
Improved/resolved: uptitrate labetalol by 400-600 mg and/or nifedipine by 30mg
Still present: recommend same day clinic visit
[1] Patient to take BP twice a day
[2] Symptoms:
Persistent headache
Scotomata
RUQ pain
[3] Or triage

Figure S1 Blood pressure management if not on antihypertensive medication

Figure S2 Blood pressure management if on antihypertensive medication

Supplement 3 Patient Communications via Gateway Portal

Patient welcome message:

Dear [Patient Name],

We are thrilled to welcome you to the Home Safe program, a dedicated initiative designed to monitor and support postpartum women like yourself in managing hypertension. Your health and well-being are of utmost importance to us, and we are committed to providing you with the highest level of care and support.

Our team of experienced healthcare professionals is here to guide you through this journey, ensuring you receive personalized and attentive care tailored to your unique needs. Throughout your time with us, you can expect:

1) Regular Monitoring: Please send in your BP readings once a day via the flowsheet on patient gateway (found under Menu -> My Record -> Track My Health) or by replying to this message. Our team will closely monitor your daily BPs, provide timely feedback, and adjust your BP medications as needed. Here is a link to a video on how to take your blood pressure: https://youtu.be/p9UEDv6nvwU?feature=shared

2) Collaborative Care: We work in tandem with your primary obstetric provider who has been informed of your enrollment in Home Safe to ensure a seamless and comprehensive approach to your well-being.

3) Open Communication: Feel free to reach out with any questions, concerns, or updates via the patient gateway. We are here to listen and provide the assistance you need.

Warm regards,

[PHC Name]
Population Health Coordinator, Obstetrics & Gynecology

Day 2 reminder:

Dear [Patient name],

Increased blood pressure can continue even after delivery, and we are here to help you manage your blood pressure through these first few weeks at home. We have yet to receive your readings. To enter your blood pressures, please use the flowsheet found under Menu -> My Record -> Track My Health. Please reach out with any questions or concerns!

Day 4 reminder:

Dear [Patient name],

I hope all is well! As you settle into home post-delivery, please remember your own recovery is important too. This is a reminder to send in your BP readings via the flowsheet on patient gateway (Menu -> My Record -> Track My Health) or by replying to this message. Please reach out with any questions or concerns!

Logging twice a week:

Hello [Patient name]

Thank you for logging your blood pressures in Patient Gateway and taking your blood pressure medication.

Your blood pressures have been normal on your current regimen, but we should still monitor you for further changes. Many people need further medication dose changes until 6 weeks postpartum

At this point it in your postpartum course, you can check your blood pressure twice a week. A common strategy is to pick 2 days of the week and check it on those days. (For example, checking blood pressures every Tuesday and Thursday).

Please respond to this message or call the office with any questions.

The Homesafe Team
617-XXX-XXXX

Discharge message:

Dear [Patient name]

Your blood pressures have been mostly at goal {"without antihypertensive medications"} OR "on your current dose of medications"}, and you have done an excellent job of sending them in! At this point, it is safe for you to stop sending us your blood pressures and to follow up with your provider as scheduled.

Thank you so much for participating in this program, and do not hesitate to contact us or your primary provider with any questions or concerns!

Your next visits are as scheduled:

Future Appointments

Date	Time	Provider	Department	Center

Best,
The Home Safe Team
